# mSphere of Influence: the Key Role of Neutrophils in Tuberculosis and Type 2 Diabetes Comorbidity

**DOI:** 10.1128/mSphere.00251-21

**Published:** 2021-05-28

**Authors:** Anne E. Mayer Bridwell

**Affiliations:** a Department of Molecular Microbiology, Washington University School of Medicine, Saint Louis, Missouri, USA

**Keywords:** *Mycobacterium tuberculosis*, NETosis, diabetes, neutrophils, tuberculosis

## Abstract

Annie Mayer Bridwell works in the field of tuberculosis pathogenesis from the host perspective. She is fascinated by comorbidities, and in this paper, she reflects on three publications that shaped her model of neutrophil-centric pathology in tuberculosis and type 2 diabetes comorbidity. She explains that “Systems immunology of diabetes-tuberculosis comorbidity reveals signatures of disease complications” (C. A. Prada-Medina, K. F. Fukutani, N. Pavan Kumar, L. Gil-Santana, et al., Sci Rep 7:1999, 2017, https://doi.org/10.1038/s41598-017-01767-4) led her to consider neutrophils as a central immunological player in comorbid patients. “Type I IFN exacerbates disease in tuberculosis-susceptible mice by inducing neutrophil-mediated lung inflammation and NETosis” (L. Moreira-Teixeira, P. J. Stimpson, E. Stavropoulos, S. Hadebe, et al., Nat Commun 11:5566, 2020, https://doi.org/10.1038/s41467-020-19412-6) and “Diabetes primes neutrophils to undergo NETosis, which impairs wound healing” (S. L. Wong, M. Demers, K. Martinod, M. Gallant, et al., Nat Med 21:815–819, 2015, https://doi.org/10.1038/nm.3887) then shed light on neutrophil extracellular trap (NET) formation as a common pathological feature of dysregulated neutrophils in tuberculosis and diabetes, respectively. Together, these works laid the foundation for Dr. Mayer Bridwell's interest in metabolic regulation of NETosis during TB infection and diabetes comorbidity.

## COMMENTARY

I am fascinated by infectious disease and pathogenicity in the complex, highly confounded context of the whole patient. This requires consideration of both environmental risk factors (e.g., socioeconomic considerations) and the patient’s biological and clinical milieu. Our bodies do not exist in a static state; biology does not occur in a vacuum. Thus, one of my longstanding interests is comorbidities, and one of my career objectives is to ask experimental questions that lead to empirical, mechanistic dissection of complex disease dynamics.

Diabetes mellitus (DM) significantly increases the risk of developing active tuberculosis (TB) disease, and it doubles the risk of death and poor treatment outcomes in TB patients (1). While diabetes increases the risk of many infections and their complications (2), the type 2 diabetes mellitus (T2DM)/TB comorbidity particularly interests me because of the high global prevalence of each disease, their overlapping geographical distributions, and the complex factors that contribute to high case numbers of each disease in large expanses of the world. In 2017, approximately 462 million individuals were affected by T2DM (3), and in 2018, there were approximately 10 million new cases of TB (4). Further, 80% of T2DM is found in low- and middle-income countries and in regions in which TB remains endemic (1).

The immunological mechanisms behind TB susceptibility in diabetic patients are not completely understood. I have selected three papers that are each impactful in their own right, and together they build a compelling argument that neutrophils are a key component perturbed in patients with both DM and TB (TBDM) (5), and that neutrophil infiltration and NETosis play a central role in severe outcomes associated with TB (6) and T2DM (7) separately.

“Systems immunology of diabetes-tuberculosis comorbidity reveals signatures of disease complications” by Prada-Medina et al. (5) took a global, systems approach to compare clinical data, plasma cytokines and growth factors, and whole-blood gene expression among healthy patients, diabetic patients, and diabetic or nondiabetic TB patients in India, which has among the highest numbers of cases of both TB and T2DM (TBDM) in the world (4). Prada-Medina et al. performed Bayesian networking across numerous clinical and immunological variables and identified neutrophils as the inflammatory nexus between DM and TB. While it is well established that neutrophilic inflammation is associated with active TB disease, this study demonstrated that the association is amplified in DM comorbid patients. Additionally, Prada-Medina et al. characterized neutrophils and type I interferons (IFN) as central players in transcriptional changes in TBDM patients. The impact of neutrophil and interferon alterations on bacterial burden and other downstream immune perturbations remains an important open question. Many diabetic patient phenotypes were exacerbated in TBDM patients; thus, Prada-Medina et al. propose amplification of diabetic complications during TB disease as a mechanism underlying increased early mortality in TBDM versus DM patients.

Given that Prada-Medina et al. identified neutrophils as a principal contributor to inflammatory perturbations in TBDM patients, it follows that my thinking has been greatly impacted by two papers that identify overlapping mechanisms of neutrophil infiltrate-induced damage in TB (6) and DM (7) disease individually. While neutrophils have emerged as key culprits in TB pathogenesis and their infiltration into Mycobacterium tuberculosis-infected lungs is associated with poor outcomes (8–11), less is known about the specific mechanisms of neutrophil-mediated susceptibility during M. tuberculosis infection and how this might be impacted by T2DM. In their exciting work “Type I IFN exacerbates disease in tuberculosis-susceptible mice by inducing neutrophil-mediated lung inflammation and NETosis,” Moreira-Teixeira et al. (6) identified the presence of neutrophil extracellular traps (NETs) in the lungs of multiple M. tuberculosis-susceptible mouse models as well as in necrotic lung lesions of TB patients. Though NETs were originally identified as a mechanism of antimicrobial defense, they also cause damaging inflammation and modulate immune responses. Moreira-Teixeira et al. linked lung-damaging NETosis to upstream type I IFN signaling based on transcriptional analysis. TB susceptibility in mice was rescued in animals with neutrophils lacking the type I IFN receptor, IFNAR, suggesting that type I IFN primes neutrophils for NET formation. This echoes the neutrophil and type I IFN transcriptional phenotype that Prada-Medina et al. highlighted in TBDM patients. Additionally, it raises a mechanistic question: does TBDM coinfection result in increased or sustained type I IFN production that leads directly to pathological NETosis?

The third paper that has influenced my thinking on the immunological mechanisms behind poor outcomes in TB-T2DM comorbidity is “Diabetes primes neutrophils to undergo NETosis, which impairs wound healing” by Siu Ling Wong et al. (7). This foundational paper was the first to describe enhanced NETosis among neutrophils from diabetic patients. It established that neutrophils exposed to high glucose, neutrophils from diabetic patients, and neutrophils from a diabetic mouse model are activated, overproduce NETs, and these NETs delay wound healing in mice. These wound healing findings suggest that diabetic NET formation could have negative implications for healing M. tuberculosis-induced lung pathology as well. Given the type I IFN and neutrophil transcriptional changes that Prada-Medina et al. identified in TBDM patients and Moreira-Teixeira et al.’s evidence of type I IFN-driven NET-associated susceptibility in M. tuberculosis-infected mouse models, this 2015 Wong et al. publication completes the diabetes arm of an additive model implicating heightened NET formation as a significant contributor to poor outcomes in comorbid mice and patients.

In combination, these three publications demonstrate the powerful strategy of using hypothesis-building approaches in combination with mechanistic immunology studies to build new models for follow-up by hypothesis-driven experimentation. Our rapidly expanding access to large, descriptive biological data sets can inform our investigations of complex disease states by revealing common themes between clinical conditions and across biological perturbations. In this case, I applied Prada-Medina et al.’s systems biology findings to the results of Moreira-Teixeira et al. and Wong et al. to build a NETosis-centric model of increased pathology in patients with TB and T2DM comorbidity. Metabolic dysregulation of immune function during infection is a theme I plan to pursue throughout my career.
